# A New Deep Learning-based Dynamic Paradigm Towards Open-World Plant Disease Detection

**DOI:** 10.3389/fpls.2023.1243822

**Published:** 2023-10-02

**Authors:** Jiuqing Dong, Alvaro Fuentes, Sook Yoon, Hyongsuk Kim, Yongchae Jeong, Dong Sun Park

**Affiliations:** ^1^ Department of Electronic Engineering, Jeonbuk National University, Jeonju, Republic of Korea; ^2^ Core Research Institute of Intelligent Robots, Jeonbuk National University, Jeonju, Republic of Korea; ^3^ Department of Computer Engineering, Mokpo National University, Muan, Republic of Korea

**Keywords:** plant disease detection, incremental learning, open-world detection, out-of-distribution detection, dynamic paradigm

## Abstract

Plant disease detection has made significant strides thanks to the emergence of deep learning. However, existing methods have been limited to closed-set and static learning settings, where models are trained using a specific dataset. This confinement restricts the model’s adaptability when encountering samples from unseen disease categories. Additionally, there is a challenge of knowledge degradation for these static learning settings, as the acquisition of new knowledge tends to overwrite the old when learning new categories. To overcome these limitations, this study introduces a novel paradigm for plant disease detection called open-world setting. Our approach can infer disease categories that have never been seen during the model training phase and gradually learn these unseen diseases through dynamic knowledge updates in the next training phase. Specifically, we utilize a well-trained unknown-aware region proposal network to generate pseudo-labels for unknown diseases during training and employ a class-agnostic classifier to enhance the recall rate for unknown diseases. Besides, we employ a sample replay strategy to maintain recognition ability for previously learned classes. Extensive experimental evaluation and ablation studies investigate the efficacy of our method in detecting old and unknown classes. Remarkably, our method demonstrates robust generalization ability even in cross-species disease detection experiments. Overall, this open-world and dynamically updated detection method shows promising potential to become the future paradigm for plant disease detection. We discuss open issues including classification and localization, and propose promising approaches to address them. We encourage further research in the community to tackle the crucial challenges in open-world plant disease detection. The code will be released at https://github.com/JiuqingDong/OWPDD.

## 1 Introduction

Accurate and timely detection and diagnosis of plant diseases are crucial for preserving crop health and increasing agricultural productivity. However, traditional methods of plant disease detection primarily rely on skilled agricultural professionals who diagnose diseases based on visual symptoms and pathologic characteristics of pathogens. These methods suffer from limitations such as subjectivity, prolonged diagnosis time, and dependence on experienced experts (Dong et al., 2022). To address these limitations of traditional methods, plant disease detection based on image analysis and artificial intelligence has emerged as a hot research topic (Shoaib et al., 2023; Xu et al., 2023). This emerging approach utilizes images captured from various plant parts such as leaves and stems, followed by computer algorithms for image analysis and recognition, enabling automated detection and diagnosis of plant diseases. This method not only enhances the accuracy and efficiency of detection but also allows non-experts to participate in plant disease monitoring and diagnosis (Panchal et al., 2023).

**Figure 1:**
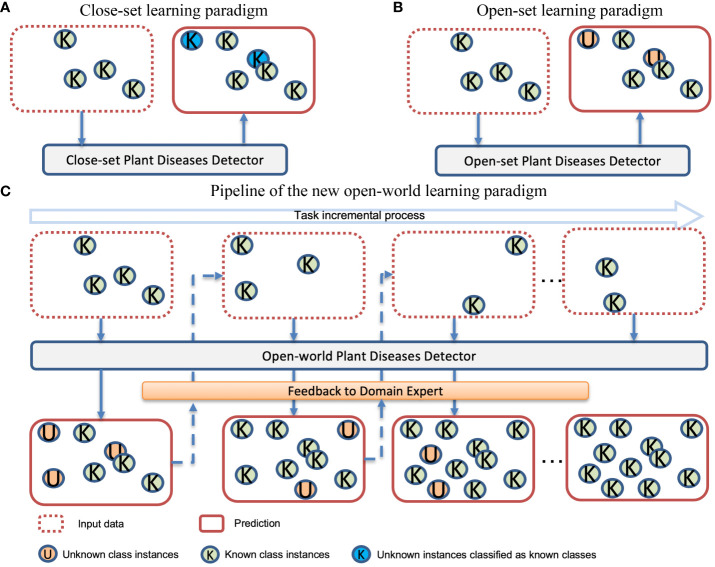
Comparison of three different learning paradigms. **(A)** Closed-set-based models detect unknown diseases as known diseases; **(B)** Open-set based models can detect unknown diseases but do not learn them; **(C)** Open-world detector learns the known diseases and also autonomously detects unknown diseases. The identified unknown diseases are then provided as feedback to domain experts, who annotate these newly discovered labels. This valuable information is incorporated into the model during subsequent tasks, allowing it to adaptively update itself with new knowledge.

A substantial body of published work attests to the success of deep learning in plant disease detection tasks (Fuentes et al., 2018; Li et al., 2020; Nazki et al., 2020; Singh et al., 2020; Fenu and Malloci, 2021; Fuentes et al., 2021; Qiao et al., 2022b). However, existing studies focus on fixed disease categories of specific species with all available annotations during the training phase. This training strategy is known as closed-set learning (Xiong et al., 2019). In this case, the model is more likely to classify suspicious regions as one of the categories it has already learned, rather than indicating the presence of an abnormal disease type (Du et al., 2022b). We show the potential risks associated with closed-set learning in **Figure 1A**. Note that “known classes” refer to the classes present in the training dataset, while “unknown classes” refer to the classes that exist in real-world scenarios but are either absent or unannotated in the training dataset.

In the concept of plant stress, unknown diseases may result in a large economic loss, and recognizing them is thus one of the fundamental demands (Geng et al., 2020). Therefore, unknown disease detection is more useful in most practical scenarios. The learning paradigm that can detect unknown classes is known as open-set learning (Vaze et al., 2021). **Figure 1B** illustrates the open-set learning paradigm, which allows the model to detect instances that are currently unknown to the model. Developmental psychology (Livio, 2017) has revealed that the ability to recognize the unknown is crucial for stimulating curiosity, which in turn fuels the desire to learn new things. In the open-set learning paradigm, when the model detects unknown diseases and provides feedback to domain experts, it is important for the domain experts to pay attention to these disease samples and assign them appropriate category labels. This allows the model to further learn about these new diseases.

To learn these new diseases, one naive learning strategy is to combine the new and old data together and let the model learn again. However, as the number of tasks increases, the accumulated data volume becomes significantly large, resulting in high training costs. This approach may be feasible in the short term but is not sustainable as a long-term training strategy. Another learning method is to fine-tune the old model using new data. In this way, the model will quickly adapt to the new task but there is a risk of losing the ability to detect previously known classes. This prompts us to propose a new challenge: a new paradigm should be capable of recognizing instances of unknown diseases as unknown and gradually learning these unknown categories through incremental learning. **Figure 1C** illustrates the workflow of this new paradigm.

Plant growth is a dynamic process, and plant disease dynamics are more complex than we imagined. During the plant growth cycle monitoring, unexpected diseases and pests are likely to emerge. Simultaneously, collecting all the existing plant diseases is difficult and even impossible for real-world applications (Xu et al., 2023). Given the dynamic nature of our world, the setup of open-world plant disease detection is more aligned with real-world applications compared to existing closed-set learning and open-set learning settings. Therefore, we need to introduce a new paradigm to continuously learn these unknown diseases instead of learning them all at once. In this paradigm, the model can detect unknown diseases and provide feedback to domain experts. Then, experts will label these unknown diseases. As and when more information about the identified unknown classes becomes available, the system should be able to incorporate them into its existing knowledge base. This iterative learning process will cycle throughout the model’s lifecycle. In this paper, we propose an open-world detector for plant disease detection, aiming to achieve this goal.

The key contributions of our work as follows:

1. We introduce the concept of open-world problem formulation into plant disease detection for the first time, enabling a closer simulation of real-world application scenarios. Unlike all existing plant disease detectors, it dynamically expands the learned categories and actively responds to unknown diseases.
2. We introduce an unknown-aware region proposal network (UA-RPN) and conducted pre-training on various datasets. We find that the model pre-trained on LVIS (Large Vocabulary Instance Segmentation) (Gupta et al., 2019) dataset can exhibit superior performance across different experimental setups. Additionally, we propose a class-agnostic region of interest (ROI) head, which significantly improved the recall rate for unknown classes. Interestingly, the model trained on a dataset of tomato leaf diseases could even detect diseases in paprika fruit.
3. Our method also achieves class incremental detection of plant diseases. Additionally, we discuss the open issues associated with open-world plant disease detection and provide promising solutions. We believe that this open-world and dynamically updated detection method can become a new paradigm for future plant disease detection, and we encourage the research community to explore and address these open challenges.

Section 2 provides a detailed review of the deep learning techniques employed for plant anomaly detection and existing open-set and open-world deep learning approaches. Section 3 comprehensively describes the problem formulation, methodology, and evaluation framework of the novel paradigm we have introduced. In Section 4, experimental results are presented to demonstrate the effectiveness and expandability of our proposed approach. We have observed that the proposed method achieves cross-species disease detection. Furthermore, we discuss the open challenges concerning plant disease detection in the context of open-world detection. In the final section, we provide several conclusions to guide future researchers. In summary, this work establishes the foundation for open-world detection in intelligent agriculture and advocates for increased attention to incremental learning and unknown target detection within the community.

## 2 Related works

In this section, we provide a brief overview of recent studies relevant to our proposed approach. Firstly, we delve into existing deep learning-based methods employed in plant disease detection. Furthermore, considering the limitations of the latest advancements in plant disease recognition, no previous work specifically addresses open-world detection. Consequently, we explore two closely related avenues: open-set detection and open-world detection.

### 2.1 Deep learning technics in plant disease detection

In recent years, various deep learning-based object detection algorithms have been applied in plant disease detection task (Qiao et al., 2022a; Shoaib et al., 2023). In the two-stage plant disease detection methods, Fuentes et al. (2017) first used Faster RCNN (Ren et al., 2015) to accurately locate tomato diseases and pests in a dataset consisting of 4800 images with 11 different classes. When using deep feature extractors like VGG-Net and ResNet, the mean average precision (mAP) was calculated as 88.66%. Liu and Wang (2021) suggested modifying the Faster RCNN (Ren et al., 2015) framework to automatically detect beet spot diseases by changing the parameters of the CNN model. Priyadharshini and Dolly (2023) provided a comparative investigation on tomato leaf disease detection and classification using RCNN (Girshick et al., 2014), Fast RCNN (Girshick, 2015) and Faster RCNN (Ren et al., 2015). Murugeswari et al. (2022) trained a model using 1500 images of healthy and diseased sugarcane leaves and deployed the model in an android application. Seetharaman and Mahendran (2022) proposed using a convolutional recurrent neural network for banana leaf disease detection. Alruwaili et al. (2022) proposed real-time faster region convolutional neural network (RTF-RCNN) for the real-time detection of tomato leaf diseases in video streams.

In the application of single-stage networks, Zhang et al. (2019) proposed a new method for detecting small agricultural pests by combining an improved version of the YOLOv3 algorithm with spatial pyramid pooling. This method addresses the low accuracy caused by the varying poses and scales of crop pests by applying deconvolution, oversampling, and convolution operations. Mathew and Mahesh (2022) used YOLOv5 to detect bell pepper leaf disease. Wang et al. (2022) optimized the lightweight YOLOv5 model for detecting peanut diseases. Additionally, Dong et al. (2022) evaluated the performance of different annotation strategies based on the YOLOv5 model.

During the training process, the aforementioned methods have access to all labels. However, they cannot locate and classify unknown diseases. In the task of plant disease classification, Fuentes et al. (2021) proposed an approach based on the concept of open-set domain adaptation to the task of plant disease recognition to allow existing systems to operate in new environments with unseen conditions and farms. To the best of our knowledge, there is currently no relevant work on detecting unknown diseases in plant disease detection tasks.

### 2.2 Out-of-distribution detection

The class in the training dataset refers to the ‘known class’ while a class existing in the test dataset but not in the training dataset is termed an ‘unknown class’. Determining whether inputs are out-of-distribution (OOD) is an essential building block for safely deploying machine learning models in the open world. OOD detection is crucial for ensuring the reliability and usability of systems in the real world. Hendrycks and Gimpel (2016) proposed a baseline for OOD detection that relies on softmax confidence scores. However, such methods can be influenced by overconfidence in the posterior distribution of OOD data. Liu et al. (2020) demonstrated mathematically that the softmax confidence score is a biased scoring function that is not aligned with the density of the inputs and hence is not suitable for OOD detection.

The energy-based model maps each input to a single scalar that is lower for observed data and higher for unobserved ones (Lecun et al., 2006). Liu et al. (2020) first proposed a unified framework for OOD detection using energy scores. Unlike softmax confidence scores, energy scores are theoretically aligned with the probability density of the input and are less susceptible to issues of overconfidence. Joseph et al. (2021) were the first to apply energy-based OOD detection to object detection. In this paper, we follow the setup of (Joseph et al., 2021) and maintain a validation set to learn the energy distribution of both known and unknown classes.

### 2.3 Open-world object detection

Open-world object detection is an emerging topic in computer vision and has attracted extensive attention due to its practicability in the real world. Unlike OOD tasks that only focus on the identification of unknown classes, open-world tasks require models to learn new classes and recognize old classes. This learning process is also known as incremental learning. To our best knowledge, there have been only a few relevant works published in top-tier conferences and journals (Joseph et al., 2021; Gupta et al., 2022; Wu et al., 2022; Ma et al., 2023a; Ma et al., 2023b; Zohar et al., 2023). Based on network architecture, these works can be categorized into methods based on Region Proposal Network (RPN) (Joseph et al., 2021; Wu et al., 2022; Ma et al., 2023b) and methods based on Transformer (Gupta et al., 2022; Ma et al., 2023a; Ma et al., 2023b; Zohar et al., 2023).

To endow the model with the capacity of detecting unknown objects, Joseph et al. (2021) proposed the Open World Object Detection (ORE) method, in which an unknown auto-labeling RPN is designed to generate pseudo labels for unknown instances. Gupta et al. (2022) and Zohar et al. (2023) employed an attention mechanism to score candidate bounding boxes, enhancing the network’s perception capability for unknown objects. Ma et al. (2023a) proposed a method that combines selective search and attention mechanisms to further enhance the retrieval capability for unknown objects. The underlying logic behind these methods is to enhance the proposal quality for unknown objects in order to obtain stronger weak supervision signals. However, methods based on attention mechanisms and selective search tend to be complex. Optimizing the perception capability for unknown objects through a simpler approach is indeed more desirable in practical engineering scenarios. Therefore, we improve the proposal quality of the network for unknown objects by using a pre-trained region proposal network (RPN), thereby enhancing the performance of open-world plant disease detection.

## 3 Methods

### 3.1 Challenges of real-world plant disease detection

Plant disease detection is a complex field that possesses distinct characteristics and challenges, particularly when considering the influence of diverse domains such as greenhouse conditions. Incremental learning serves as a crucial tool to address these challenges and enhance the accuracy and adaptability of disease detection systems.

#### 3.1.1 Characteristics of plant disease detection

The process of plant disease detection is marked by several unique characteristics. Unlike some other domains, plant health is influenced by an intricate interplay of factors. Variations in features across plant species, genetic diversity, and environmental conditions lead to a diverse range of disease symptoms. These symptoms can be subtle, ranging from changes in leaf color and texture to wilting and necrosis. Additionally, the progression of diseases can vary widely, making it challenging to predict the trajectory and severity of an infection.

#### 3.1.2 Challenges in diverse domains and greenhouse conditions

Diverse domains, such as greenhouse environments, introduce a set of challenges that impact plant disease detection. Greenhouses provide controlled conditions for plant growth, which can accelerate disease progression due to the close proximity of plants, regulated temperature, and humidity. The dynamic interactions between plants, pathogens, and the environment within greenhouses contribute to complex disease patterns that traditional, static models might struggle to capture. Moreover, the controlled environment can lead to rapid mutations in pathogens, adding further complexity to disease identification.

In a domain characterized by diverse symptoms, feature variations, environmental factors, and disease progression, previous models to detect plant disease can fall short. Our proposed approach, however, enables models to evolve alongside the evolving disease landscape. The adaptive nature of our approach allows models to incorporate new information, adapt to feature variations, and account for changing environmental conditions. As the disease patterns shift and pathogens mutate, incremental learning ensures that the detection system remains up-to-date and effective. This is particularly critical in greenhouse conditions, where rapid disease spread demands real-time monitoring and rapid response.

### 3.2 Problem formulation

In this section, we provide a formal definition of Open World Object Detection. In a closed-setting approach, a model is trained on a specific set of known classes and then tested on data collected from the same or similar environment such as $D_{train}^{closed-set}={\left\{\left(x_{i},y_{i}\right)\right\}}_{i=1}^{M}\subset X\;\times \;C$ , and $D_{test}^{closed-set}={\left\{\left(x_{i},y_{i}\right)\right\}}_{i=1}^{N}\subset X\;\times \;C$ , where X denotes the image samples in the dataset D , and C indicates the number of classes. However, real-world scenarios often involve new environments and the presence of unknown diseases that the model has not encountered before. Consequently, when tested on such data, the model may fail to perform accurately. In this context, the test dataset is $D_{test}^{open-world}={\left\{\left(x_{i},y_{i}\right)\right\}}_{i=1}^{N}\subset X\;\times \;$ *(C*&*U)*, where U denotes unknown classes in training phase. Therefore, in open-world disease detection, the primary target is to detect these unknown diseases.

After achieving the primary target, the model becomes capable of identifying diseases that were not part of the initial training set (unknown diseases). Our objective is for the model to learn these new classes in subsequent learning tasks while retaining its recognition ability for the classes learned int eh previous tasks. We define the initial training task as Task 1 and subsequent tasks as Task 2, Task 3, and so on. In Task 1, the training dataset, denoted as $D_{T1}$ , consists of labeled samples for a number of $C_{T1}$ disease classes. However, during the inference process, the model may encounter instances of unknown diseases that were not seen during training. To address this, the model needs to accurately locate these unknown disease types and assign them the label ‘unknown’. These unknown disease instances will be presented to domain experts for annotation and will be used for training in Task 2. In Task 2, the number of new disease classes is denoted as $C_{T2}$ . After completing Task 2, the set of known classes is updated to the previously known classes $C_{T1}$ along with the newly learned classes $C_{T2}$ . However, during the inference process, the model still may encounter unknown diseases that do not belong to the known classes $\left(C_{T1}+C_{T2}\right)$ . Therefore, in addition to detecting the known classes, the model will continue to identify unknown diseases and assign them the label ‘unknown’. These unknown disease instances will be learned in Task 3.

This cycle of updating the model’s knowledge continues throughout the entire lifecycle of the detector. In each task, the detector acquires new knowledge without forgetting the previously learned classes. This allows the model to continuously adapt and improve its detection capabilities by incorporating new information in a progressive manner.

### 3.3 Datasets and splits

After defining the open-world problem, it is necessary to search for suitable datasets to evaluate our method. In this study, we extended the tomato dataset used in previous works (Fuentes et al., 2018; Fuentes et al., 2021) to include 15 different classes, which were learned in Task 1, Task 2, and Task 3, respectively. To ensure a balanced distribution, we divided the classes equally, with 5 different classes assigned to each task. In Task 1, instances belonging to the classes of Task 2 and Task 3 were not available. Additionally, we aimed to investigate the performance of our model in cross-species training. For this purpose, we incorporated the paprika disease detection dataset (Dong et al., 2022) in Task 4. The tomato dataset originally consisted of 15 classes, while the paprika dataset contained 5 classes. To ensure the dataset’s representation of real-world scenarios and to introduce complexity, we excluded images collected in a laboratory setting. This approach prevents potential overestimation of the model’s performance and enhances the dataset’s ability to simulate real-world conditions.

For each task, we employed a random selection process to designate 20% of the integrated dataset (combining tomato and paprika data) as the validation data. This allowed us to learn the distribution of known and unknown samples within this subset. Additionally, we randomly chose 20% of the data as the test set, which was used across all tasks. Here we aim to address the question: why do we test diseases from different species together? There are several reasons for this approach. Firstly, evaluating the performance of our model on different species’ diseases allows us to assess its generalization capability across species. In real-world scenarios, plant disease detection systems encounter various species and their associated diseases. By testing different species’ diseases together, we can effectively assess how well our model handles the challenges of detecting diseases across multiple species. This includes dealing with variations in symptoms, visual appearances, and disease patterns. Such evaluation helps us gain insights into the robustness and effectiveness of our model in practical applications where encounters with a diverse range of plant species are expected. Furthermore, successful detection of diseases from different species indicates that our model has acquired solid features of diseases as a concept. It demonstrates that the model’s learning transcends species-specific information and can be effectively applied to diverse plant species. The dataset split and more specific details are presented in **Table 1**. Unless otherwise specified, the training order of all experiments in this paper follows the sequence shown in **Table 1**.

**Table 1:** Task composition and data split in the proposed open-world plant disease detection protocol.

Task sequence	Task 1	Task 2	Task 3	Task 4
Species	Tomato	Tomato	Tomato	Paprika
Training Classes	5	5	5	5
Categories	magnesium deficiency, gray mold, leaf mold, yellow leaf curl virus, physical damage	canker, plague, leaf miner, white fly, white fly egg	wilt, chlorosis virus, stress, powdery mildew, old leaf	blossom end rot, gray mold, powdery mildew, spider mite, spotting disease
Training images	3236	1728	1647	2049
Validation images	2491	2491	2491	2491
Test images	2493	2493	2493	2493
Known Classes	5	10	15	20
Unknown Classes	15	10	5	0

### 3.4 Architecture

In their study, Dhamija et al. (2020) found that two-stage networks outperform single-stage networks when it comes to detecting unknown objects. Motivated by this finding, we have chosen to implement our open-world detection model using the classic Faster RCNN (Ren et al., 2015), which is a two-stage network architecture. To enhance the representation of multi-scale features, we have incorporated the feature pyramid network (FPN) (Lin et al., 2017).

**Figure 2:**
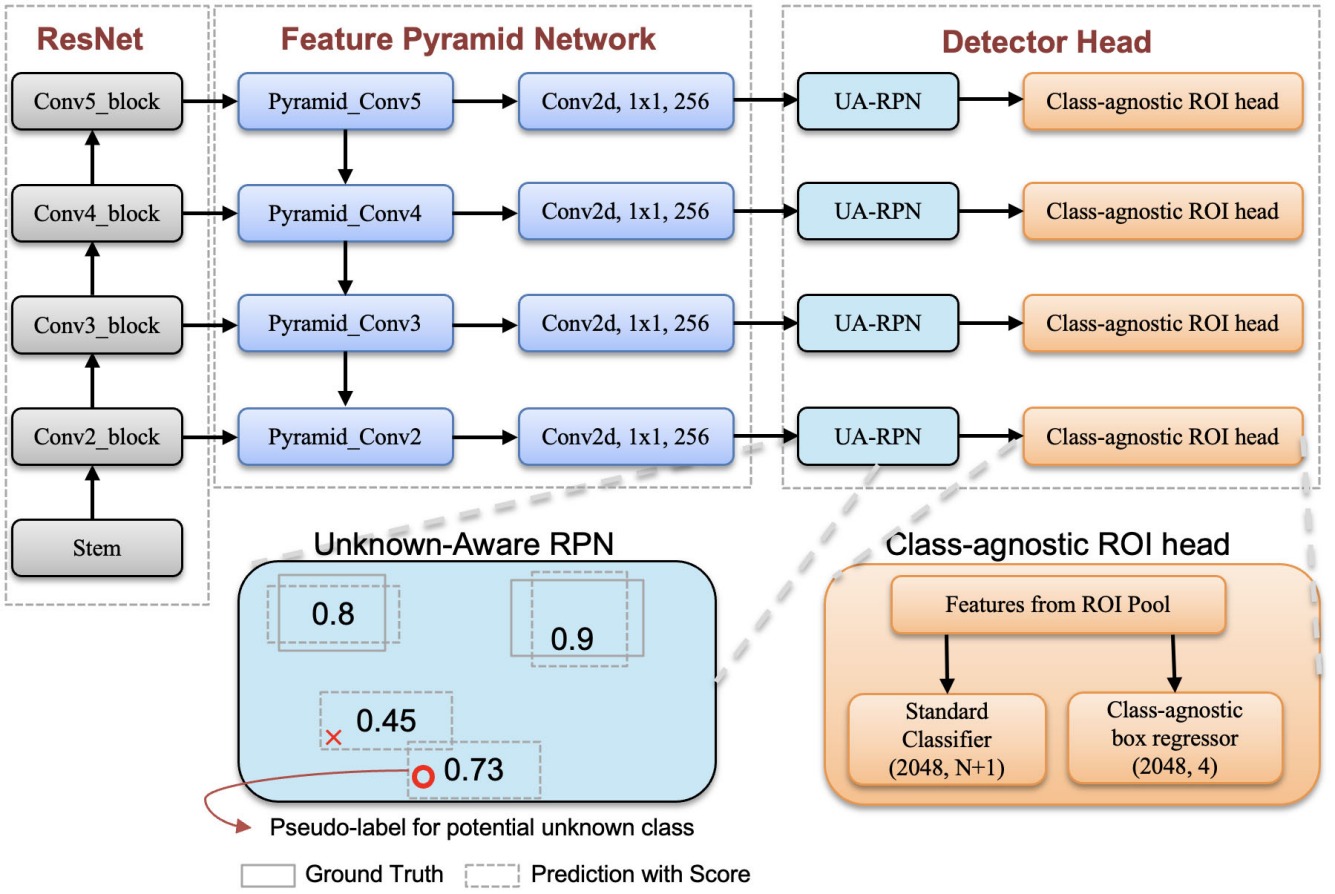
Overview of our model where ResNet and FPN are constructed following the default approach in detectron2 (Wu et al., 2019). We illustrate the unknown-aware RPN and class-agnostic ROI head in the diagram. Unknown aware RPN modifies the labels of background candidate boxes with the highest object scores to ‘unknown’. The class-agnostic head focuses on regressing bounding boxes for disease regions without considering the disease category.

In **Figure 2**, we present an illustration of the Faster RCNN with the FPN network. Please note that our method, unlike the standard Faster RCNN, can detect unknown classes. This capability is achieved through a well-trained unknown perception Region Proposal Network (RPN) and a class-agnostic localization head. The unknown perception RPN is designed for automatic labeling of unknown objects, while the class-agnostic localization head is responsible for accurately localizing these unknown objects. Each of these components is explained in detail in the following subsections, providing a coherent understanding of their roles in our model.

### 3.5 Well-trained unknowns-aware RPN

In the context of object detection tasks, the objective is to identify and localize objects of interest within an image. Traditional object detection models are typically trained on datasets that consist of known classes, assuming that all objects can be classified into predefined categories. However, real-world scenarios often present instances where the model encounters objects belonging to unknown or unseen classes.

To address the challenge of detecting unknown diseases, we introduce an additional “unknown” class during the training process. This class is assigned as a pseudo label ‘unknown’ to proposals that have a high objectness score but do not overlap with any ground-truth objects. To generate high-quality proposal boxes, we directly train the detector on the object detection dataset to obtain well-initialized parameters. A well-trained RPN can generate highly accurate proposals or candidate object regions within an image. These proposals effectively filter out cluttered or background regions, enabling the model to focus solely on relevant object proposals. This capability helps in reducing false positives and improving overall detection accuracy. Additionally, a well-trained RPN can effectively handle objects of different sizes and shapes. It learns to generate proposals that encompass objects with varying aspect ratios, ensuring comprehensive coverage of the object space. This enables the model to effectively handle novel or unseen objects, thereby enhancing its performance and robustness in open-world scenarios. We further compare the performance of different pre-trained datasets in open-world plant disease detection.

### 3.6 Class-agnostic ROI head

Locating unknown diseases is an important issue in open-world detection tasks. Standard detectors are primarily designed for localizing objects of known classes, as they employ class-specific localization methods. For instance, detectors like Faster RCNN (Ren et al., 2015) and Mask RCNN (He et al., 2017) generate class-specific bounding boxes for each known class when the proposals enter their prediction heads.

To address the localization of novel objects, we introduce a class-agnostic Region of Interest (ROI) head in our object detection models. The class-agnostic ROI head treats region-based feature extraction and classification tasks independently of specific object classes. Unlike class-specific ROI heads that are designed to predict object classes for each region, the class-agnostic ROI head focuses solely on generating accurate bounding box regression outputs without considering the object categories. This makes it well-suited for open-world object detection scenarios where unknown or novel classes may appear.

Inspired by the learned objectness (Kuo et al., 2023), we utilize class-agnostic box regression heads instead. We have observed that class-agnostic ROI heads exhibit better generalization to unseen classes during inference. They are not biased towards specific object categories, allowing the model to adapt to new classes without the need for retraining or fine-tuning. Additionally, by removing the class-specific classification branch, the overall architecture becomes simpler and more streamlined. This modification not only reduces the computational complexity and memory requirements of the model but also enables more efficient handling of unknown classes.

### 3.7 Alleviating forgetting

Catastrophic forgetting (Hayes et al., 2020) refers to the phenomenon observed in incremental learning, where a model trained on new data gradually loses or forgets the knowledge acquired from previously learned tasks or classes. This occurs when the new data heavily influences the model’s parameters, leading to the overwriting or disrupting of previously learned information. To address catastrophic forgetting, several techniques have been proposed, such as parameter isolation (Prabhu et al., 2020), regularization (Li and Hoiem, 2017), and sample replay (Rebuffi et al., 2017). These techniques reinforce the model’s memory of previous tasks or classes by incorporating previously observed samples during training. In this way, the model can maintain its performance on old tasks while learning new ones.

Sample replay is relatively straightforward compared to other techniques like parameter isolation or complex regularization strategies. It periodically included old samples in the training dataset, making integrating them into existing training pipelines easy. The simplest form of sample replay is randomly retaining training samples. This paper follows the sample replay strategy proposed by Joseph et al. (2021), which is the simplest way of sample replay. After each incremental step, a balanced set of samples is stored randomly, and the model is fine-tuned. To ensure an adequate representation of each class, we guarantee a minimum of $N_{samples}$ instances for each class in the sample set. Generally, a larger $N_{samples}$ tends to result in better fine-tuning performance (an extreme case being the use of the entire dataset). However, this contradicts the original intention of dynamic learning in an open-world setting. To ensure a fair comparison among the models, we set $N_{samples}=25$ for fine-tuning the model.

### 3.8 Evaluation metrics

We present a comprehensive evaluation protocol to assess the performance of an open-world detector in various aspects: identifying unknown classes, detecting known classes, and progressively learning new classes when labels are available for some unknown samples.

#### 3.8.1 Mean average precision score

mAP is the area under the precision-recall curve calculated for all classes. To evaluate the detection performance of known classes, we utilize the standard mean average precision (mAP) metric with an intersection over union (IoU) threshold of 0.5 [mAP@50, consistent with the existing literature (Joseph et al., 2021; Gupta et al., 2022; Wu et al., 2022; Ma et al., 2023a; Ma et al., 2023b; Zohar et al., 2023)].


(1) $$\text{AP}=\frac{1}{11}\sum_{\text{r}\in \left[0,0.1,\ldots ,0.9,1\right]}\text{P}\left(\text{r}\right)$$

(2) $$P\left(r\right)=\underset{\tilde{\text{r}}:\tilde{\text{r}}\geq \text{r}}{\text{max}}\text{p}\left(\tilde{\text{r}}\right)$$

where, P(r) is the maximum precision for any recall values greater than r, and $p\left(\tilde{r}\right)$ is the measured precision at recall $\tilde{r}$ . Since the problem setting of open-world detectors is different from that of standard detectors, there are three forms of mAP, which are current classes mAP, previous classes mAP, and known classes mAP.

#### 3.8.2 Unknown recall

We employ recall as the main metric for unknown object detection instead of the commonly used mAP. This is because all possible unknown object instances in the dataset are not annotated. Unknown recall is widely used in open-world object detection (Gupta et al., 2022; Ma et al., 2023a; Ma et al., 2023b; Zohar et al., 2023).


(3) $$\text{U}-\text{R}=\frac{\text{TPU}}{\text{AU}}$$

where, TPU is the true positive of unknown instances, and AU denotes all unknown instances for the current task.

#### 3.8.3 Absolute open-set error

In addition, we employ the Absolute open-set error (A-OSE) (Miller et al., 2018) metric to report the number of unknown objects that are misclassified as any of the known classes. This metric implicitly measures how effective the model is in handling unknown objects.

To facilitate readability, we use the abbreviations listed in **Table 2** to denote the evaluation metrics. The metrics include Unknown Recall and A-OSE, which assess the performance of the unknown classes, and Mean Average Precision (mAP), which evaluates the model’s ability to detect the known classes. By employing these metrics, we can comprehensively evaluate and compare the model’s performance across both known and unknown classes, providing a comprehensive assessment of its detection capabilities.

**Table 2:** Abbreviation and meaning of the evaluation metrics.

Abbreviation	Meaning	Others
P	mean average precision score of previous task classes	↑
C	mean average precision score of current task classes	↑
K	mean average precision score of all known classes	↑
A	Absolute open-set error	↓
U-R	Unknown-Recall	↑

Arrows indicate expected trends. Up means that the larger the value, the better, and vice versa.

## 4 Results

### 4.1 Implementation details

In the training task sequence, the model can only access the data from the current task. Known classes are defined as the classes in the current task as well as the previous tasks, while other classes are defined as unknown classes. For each image, the model generates only one unknown instance. We adopted the contrastive clustering loss proposed by ORE (Joseph et al., 2021) and used stochastic gradient descent to optimize the model, with a batch size set to 4. For each training task, we iterated 18,000 times, and for each fine-tuning task, we iterated 4,000 times. We used ResNeXt101 (Xie et al., 2017) as the final backbone. The entire training process for the project, conducted on 4 NVIDIA GeForce RTX 3090 GPUs, was completed in less than 12 hours. For more details, please refer to our code.

### 4.2 Overall results

**Table 3** compares our method with Faster RCNN (Ren et al., 2015) and ORE (Joseph et al., 2021) using the proposed open-world evaluation protocol. The 1-3 row in **Table 3** showcases the result obtained by the standard Faster-RCNN. Note that we used the ResNet50 backbone on the ImageNet1K dataset as a pretraining backbone. We provide a brief overview of the training approach for Faster RCNN. Row 1: We trained Faster-RCNN using a static closed-set training strategy for a fair comparison. As anticipated, Faster-RCNN trained with the closed-set strategy demonstrated optimal results in closed-set evaluation metrics, because the model retrained with all known datasets for each task. However, the model’s focus remains limited to known categories, incapable of identifying unknown targets, which contradicts the open-world setting. This experimental set allows researchers to grasp the upper-performance limits of the model in known-category recognition tasks. Hence, we employ ‘Upper’ to denote the results of this experiment. Row 2: We trained the standard Faster-RCNN on Task 1, followed by Task 2, Task 3, and Task 4. After completing each task, the model’s performance was evaluated through testing. In this scenario, the model was also unable to identify unknown diseases. We observed a significant decline in detection performance for previous classes during subsequent task learning with the standard Faster RCNN, which indicates that new knowledge quickly replaced old knowledge throughout the training process. In contrast, our method can successfully detect unknown classes and continuously learn new categories without the need to train from scratch. Row 3: We employed a sample replay strategy to train Faster-RCNN dynamically. This experimental set allows researchers to understand how much sample replay preserves the model’s memory capabilities. We denote the results of this experiment as ‘Faster-RCNN*’ in **Table 3**.

**Table 3:** Overall results of our method compared with the baseline approach.

Methods	Task 1			Task 2					Task 3					Task 4			Parameter
	C	A	U-R	P	C	K	A	U-R	P	C	K	A	U-R	P	C	K	
Upper	63.9	2044	–	69.20	60.54	64.91	1706	–	67.06	48.66	60.93	705	–	63.88	84.40	69.02	33M
Faster RCNN	63.9	2044	–	6.6	42.9	24.8	–	–	3.6	25.5	10.9	–	–	1.8	54.3	14.9	33M
Faster RCNN*	63.9	2044	–	62.8	40.3	51.6	2386	–	50.7	32.2	44.5	1226	–	44.7	62.2	49.1	33M
ORE	63.5	2002	14.2	62.6	39.1	50.9	2303	4.7	48.2	31.0	42.5	1228	5.1	42.9	62.7	47.9	33M
Ours (a)	65.4	2124	22.6	63.2	43.2	53.2	2190	12.4	50.6	29.3	43.5	1572	13.1	44.5	61.5	48.8	41M
Ours (b)	60.7	1827	24.0	63.1	46.3	54.7	3144	8.9	52.4	28.2	44.4	1638	**38.3**	45.3	67.0	50.7	41M
Ours (c)	62.4	1932	**25.3**	62.7	44.9	53.8	1961	13.0	52.7	29.5	45.0	1211	13.0	47.1	67.1	52.1	41M
Ours (d)	65.2	1880	23.8	63.2	47.0	55.1	1960	**13.7**	54.4	31.3	46.7	1418	15.7	46.8	66.9	51.8	41M
Ours*	**66.3**	**1559**	22.7	**64.0**	**48.9**	**56.5**	**1854**	**13.4**	**54.5**	**35.8**	**48.3**	**1123**	10.8	**48.2**	**70.0**	**53.6**	104M

For the notation of evaluation metrics, please refer to **Table 2**. Bold font represents the optimal results. “Upper” represents training using the entire dataset, which theoretically serves as the performance upper bound. “Faster RCNN*” represents training using a dynamic paradigm.

Ours* is a larger model than ours (d), other settings are the same.

'-' indicates that the evaluation metric is not applicable to the current experimental setup.

**Figure 3:**
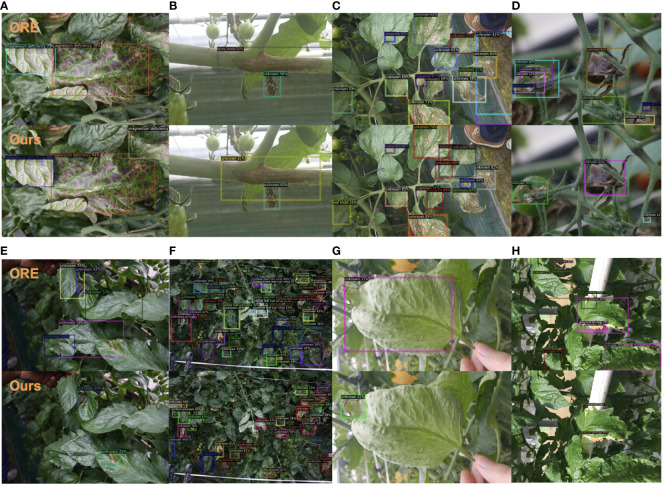
Visualization results comparison between ORE and our model, both trained on Task 1. We present eight pairs of examples **(A-H)**. Best view in color.

Furthermore, our four variants, labeled as Ours (a), Ours (b), Ours (c), and Ours (d) utilized the ResNet-50-FPN backbone, but were pretrained on different datasets. Specifically, Ours (a) used Imagenet-1k (Deng et al., 2009), Ours (b) used COCO (Lin et al., 2014), Ours (c) used Object-365-v2 (Shao et al., 2019), and Ours (d) used the LVIS (Gupta et al., 2019) dataset. These experiments demonstrate that our method consistently outperforms the ORE (Joseph et al., 2021) baseline across all evaluation metrics. Additionally, we explored the ResNeXt101(Xie et al., 2017)architecture, an extension of ResNet, which introduced cardinality to enhance feature representation, making it potentially more powerful in capturing complex patterns and achieving better performance compared to ResNet101. To further improve the model’s performance, we trained the ResNeXt-101-FPN on the LVIS dataset. The final row in the table shows the results of our method using the ResNeXt-101-FPN backbone pre-trained on the LVIS dataset, denoted as Ours* in **Table 3**. Note that A-OSE scores and unknown recalls cannot be measured for Task 4 because of the absence of unknown ground truths. For a visual comparison with the baseline, we present the detection results for Task 1 in **Figure 3**. Our model outperformed ORE in terms of known disease detection, demonstrating higher accuracy in **Figures 3A, B**. Furthermore, when it comes to unknown diseases, our model excelled in reducing false positives as seen in **Figures 3C, D**. Additionally, our model achieved precise localization for unknown diseases, as evident in **Figure 3E–H**.

**Figure 4:**
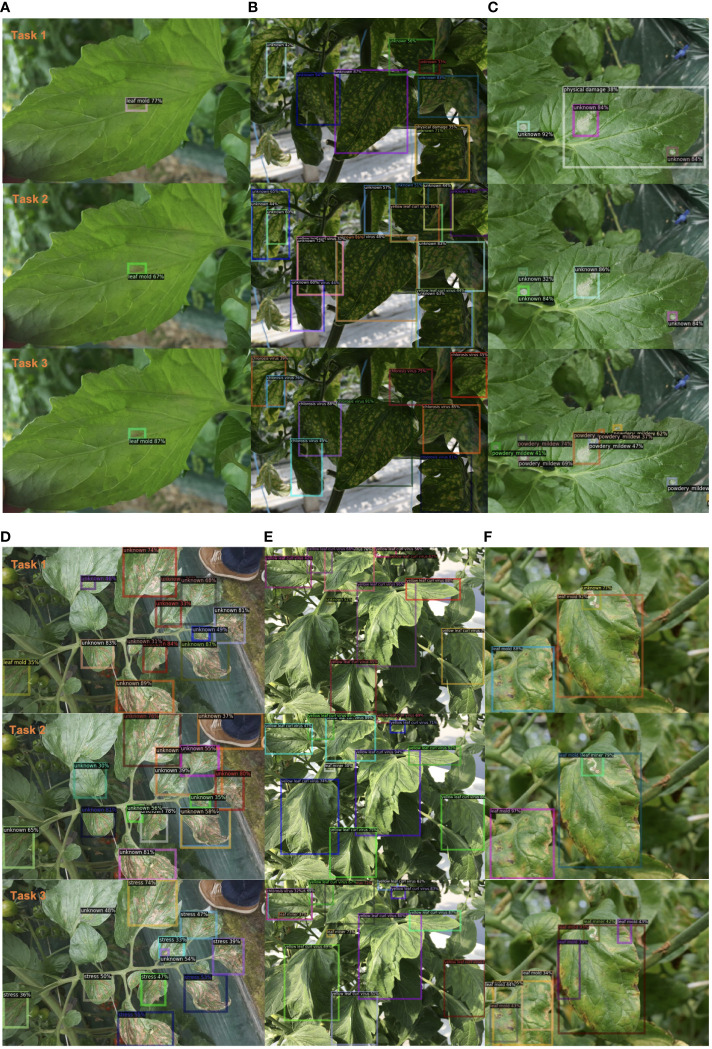
Qualitative results of our method on example images from our plant disease dataset. We present six groups of examples **(A-F)** from Task 1 to Task 3. Best view in color.

Furthermore, in **Figure 4**, we present additional qualitative results, showcasing a batch of images that were tested on our model across three tasks. Case A and Case E highlight the model’s ability to remember previously learned classes, accurately classifying and locating diseases learned in Task 1. Cases B, C, and D demonstrate the model’s capability to detect unknown diseases and progressively learn them. Although these instances were unknown in Task 1, the model gradually learned them in Task 2 and Task 3. Additionally, we include a set of failed cases where the model started to exhibit confusion in localizing old classes as new knowledge is introduced. These challenges will be addressed in future studies.

### 4.3 Ablation experiment

To analyze the individual contributions of each component in our method, we conducted meticulous ablation experiments, and the results are presented in **Table 4**.

**Table 4:** Ablation results. PTD and CAH denote pre-trained dataset and class-agnostic head, respectively.

Architecture	PTD	CAH	Task 1			Task 2					Task 3					Task 4		
			C	A	U-R	P	C	K	A	U-R	P	C	K	A	U-R	P	C	K
R-50-C4	IN1k	x	63.5	2002	14.2	62.6	39.1	50.9	2303	4.7	48.2	31.0	42.5	1228	5.1	42.9	62.7	47.9
R-50-FPN	IN1k	x	67.6	2173	14.7	63.6	42.9	53.3	2176	13.2	51.5	30.0	44.3	1688	7.8	44.8	65.7	50.0
R-50-FPN	IN1k	√	65.4	2124	22.6	63.2	43.2	53.2	2190	12.4	50.6	29.3	43.5	1572	13.1	44.5	61.5	48.8
R-50-FPN	COCO	x	59.0	1906	12.4	60.3	44.9	52.6	2075	10.4	52.9	28.1	44.6	1468	4.1	45.6	67.3	51.0
R-50-FPN	COCO	√	60.7	1827	24.0	63.1	46.3	54.7	3144	8.9	52.4	28.2	44.4	1638	38.3	45.3	67.0	50.7
R-50-FPN	Object365	x	62.0	2066	10.5	62.1	47.1	54.6	1971	9.8	53.2	36.4	47.6	1300	5.1	47.8	69.1	53.1
R-50-FPN	Object365	√	62.4	1932	25.3	62.7	44.9	53.8	1961	13.0	52.7	29.5	45.0	1211	13.0	47.1	67.1	52.1
R-50-FPN	LVIS	x	64.6	2036	13.0	61.9	46.9	54.4	2156	13.5	55.0	32.9	47.6	1566	9.1	47.1	69.1	52.6
R-50-FPN	LVIS	√	65.2	1880	23.8	63.2	47.0	55.1	1960	13.7	54.4	31.3	46.7	1418	15.7	46.8	66.9	51.8

IN1k denotes the Imagenet-1k dataset. For the notation of evaluation metrics, please refer to **Table 2**.

'√' and 'x' respectively indicate model with or without the class-agnostic head.

#### 4.3.1 Backbone

We compared the FPN module with the C4 module on ResNet-50 (Row 1 and Row 2). The inclusion of FPN significantly enhanced the model’s learning ability and memory capacity, as evidenced by improved performance in Task 1 (63.55% vs. 67.67%) and Task 3 (48.27% vs. 51.50%). Based on this observation, all subsequent experiments were performed using ResNet with FPN as the backbone network instead of the C4 structure.

#### 4.3.2 Class-agnostic head

Our class-agnostic head played a crucial role in the model’s performance. By not assigning specific class labels to detected objects, the class-agnostic head enabled the model to treat all objects as potential unknown classes. This means that if a detected object does not match any known class, it is more likely to be classified as an unknown object rather than misclassified into a known class. Consequently, the class-agnostic head improved the model’s ability to recognize and recall unknown objects, thus enhancing overall performance in open-world scenarios. **Table 4** demonstrates that the class-agnostic head significantly improved the recall of unknown classes across different pretraining data. Moreover, **Table 5** indicates that the class-agnostic head remains effective even when used with larger networks.

**Table 5:** Results of our method using larger model.

Architecture	PTD	CAH	Task 1			Task 2					Task 3						Task 4		
			C	A	U-R	P	C	K	A	U-R	P	C	K	A	U-R	P		C	K
R-50-C4	IN1k	x	63.5	2002	14.2	62.6	39.1	50.9	2303	4.7	48.2	31.0	42.5	1228	5.1	42.9		62.7	47.9
R-50-FPN	IN1k	x	67.6	2173	14.7	63.6	42.9	53.3	2176	13.2	51.5	30.0	44.3	1688	7.8	44.8		65.7	50.0
R-50-FPN	LVIS	√	65.2	1880	23.8	63.2	47.0	55.1	1960	13.7	54.4	31.3	46.7	1418	15.7	46.8		66.9	51.8
R-101-FPN	LVIS	√	64.8	1815	24.0	65.4	44.1	54.7	2448	9.3	54.2	34.5	47.6	1418	7.6	48.3		71.8	54.1
X-101-FPN	LVIS	√	66.3	1559	22.7	64.0	48.9	56.5	1854	13.4	54.5	35.8	48.3	1123	10.8	48.2		70.0	53.6

For a fair comparison, we show the results of the baseline (R50-C4) and Resnet-50 with FPN (R50-FPN).

'√' and 'x' respectively indicate model with or without the class-agnostic head.

#### 4.3.3 Pretraining datasets

In order to investigate the influence of different pre-training datasets on our model, we conducted a series of experiments as outlined in **Table 6**. Our findings reveal that the model trained on the Imagenet-1k dataset exhibited better performance on the initial tasks. However, as the tasks progressed, this advantage gradually diminished. On the other hand, the model trained on the LVIS dataset showed an advantage in terms of unknown recall, with no significant drop in performance (mAP) for known class detection. Similarly, the model trained on the COCO dataset exhibited a similar trend, albeit with slightly lower performance.

**Table 6:** Results of different pre-training data in the open-world disease detection tasks.

(A)																		
Architecture	PTD	CAH	Task 1			Task 2					Task 3					Task 4		
			C	A	U-R	P	C	K	A	U-R	P	C	K	A	U-R	P	C	K
R-50-FPN	IN1k	x	**67.6**	2173	**14.7**	**63.6**	42.9	53.3	2176	13.2	51.5	30.0	44.3	1688	7.8	44.8	65.7	50.0
R-50-FPN	COCO	x	60.9	**1950**	11.8	59.4	44.0	51.7	2238	9.4	51.5	27.6	43.5	1731	1.3	44.9	66.3	50.2
R-50-FPN	Object365	x	62.0	2066	10.5	**62.1**	**47.1**	**54.6**	**1971**	9.8	53.2	**36.4**	**47.6**	**1300**	5.1	**47.8**	**69.1**	**53.1**
R-50-FPN	LVIS	x	64.6	2036	13.0	61.9	46.9	54.4	2156	13.5	**55.0**	32.9	47.6	1566	**9.1**	47.1	69.1	52.6
(B)																		
R-101-FPN	IN1k	x	**66.7**	2042	**14.9**	63.2	44.0	53.6	**2038**	**13.2**	52.1	31.0	45.0	1538	3.7	44.9	62.4	49.2
R-101-FPN	COCO	x	61.5	**1842**	10.5	61.9	45.1	53.5	2367	6.7	52.1	28.4	44.2	1538	2.7	45.5	69.3	51.4
R-101-FPN	Object365	x	62.6	2077	11.6	62.5	44.1	53.3	2035	10.0	53.7	31.8	46.4	1546	**8.2**	47.7	65.4	52.1
R-101-FPN	LVIS	x	64.1	1910	12.7	**63.8**	**45.9**	**54.8**	2338	5.5	**53.7**	**32.2**	**46.6**	**1583**	4.8	47.6	**72.1**	**53.8**
(C)																		
X-101-FPN	IN1k	x	**69.0**	1832	**18.1**	**65.0**	46.4	55.7	1894	8.7	52.8	37.3	47.6	1231	1.1	45.9	66.8	51.1
X-101-FPN	COCO	x	58.0	**1594**	11.5	60.6	47.4	54.0	2029	4.1	52.5	33.5	46.2	1542	6.3	45.7	66.7	51.0
X-101-FPN	Object365	x	61.0	1774	15.9	63.6	47.3	55.5	**1864**	**9.6**	51.8	33.6	45.7	**1140**	**7.8**	46.5	69.1	52.2
X-101-FPN	LVIS	x	63.5	1668	12.7	62.9	**48.4**	55.6	1907	5.5	**54.4**	**36.1**	**48.3**	1398	4.5	**48.8**	**71.8**	**54.6**

We compared three different network models: ResNet50 with FPN, ResNet101 with FPN, and ResNetX101 with FPN. Bold font indicates the best results among each comparison group.

'x' denotes the model without the class-agnostic head.

We attribute the benefits brought by the LVIS-based pre-trained models to two main factors. Firstly, the consistency of pre-training objectives played a significant role. The LVIS-based pre-trained models utilize training objectives that align closely with the target detection task, encompassing multi-label classification and bounding box regression. In contrast to ImageNet pre-trained models, these objectives are better suited for the target detection task, resulting in improved performance. Secondly, the richness of the data is a contributing factor. LVIS encompasses over 1,200 categories, whereas COCO only includes 80 categories. The significantly larger number of categories in LVIS provided a more diverse and comprehensive representation of objects across various domains. Consequently, this allowed the LVIS-based pretrained models to learn more comprehensive features and contextual information for different categories. Based on these observations, we argue that the pre-training model based on LVIS exhibited greater potential for subsequent tasks due to the alignment of training objectives and the broader representation of object categories.

Furthermore, we also trained and released these three models on the Object365 dataset using the Detectron2 framework. The Object365-v2 dataset (Shao et al., 2019) contains nearly 2 million images with over 10 million annotated bounding boxes. In terms of scale, Object365-v2 contains a greater number of instances compared to LVIS. However, we observed that pre-trained on the Object365-v2 dataset significantly boosts the performance of the COCO dataset in open-world evaluation settings, but its performance on plant disease datasets is slightly lower than the model pre-trained on LVIS dataset. As a result, we opted for the LVIS-based pre-trained model as the final choice for our work. Please note that fine-tuning the COCO dataset results using the Object365-v2 dataset is not the focus of this paper. We presented these results in our code repository.

Additionally, we performed experiments using larger models to enhance the performance of our model, as presented in **Table 5**. It was challenging to improve all performance metrics across all tasks simultaneously. However, in general, employing larger models, leveraging well-pretrained Region Proposal Networks (RPNs), and incorporating class-agnostic heads tended to yield better results.

### 4.4 Sensitivity analysis on training order

In the context of incremental learning tasks, the order in which tasks are presented to the model can significantly impact its performance and the overall learning process. The learning sequence plays a crucial role in addressing challenges such as knowledge forgetting, conflicting information, and fluctuations in performance. Recognizing the importance of the learning order, we conducted an investigation to understand the model’s sensitivity to different training sequences.

By analyzing the results in **Table 3**, we observed that Faster RCNN achieved the highest performance on Task 1 and the lowest on Task 3 when detecting tomato diseases. This observation led us to infer that Task 1 might be relatively simpler, while Task 3 could pose more challenges in disease detection.

Following the principle of human learning from easy to difficult, we believe that the model should start learning from simple tasks. Therefore, in previous experimental settings, the default learning order was from Task 1 to Task 3. After learning the diseases of one species, the model continued to learn the cross-species detection task (Task 4).

However, to explore the sensitivity of our research model to the training order, we decided to deviate from the default sequence and adopt a different approach. We opted to initiate the learning process with the more difficult Task 3. By doing so, we aimed to observe how the model adapts and performs when confronted with the most challenging task from the start. Therefore, in this study, we rearranged the task sequence as follows: Task 3, Task 2, Task 1, and finally, Task 4.

This alternative task sequence enabled us to investigate the model’s ability to learn and transfer knowledge in a non-conventional order, offering insights into its adaptability and potential for early tackling of more complex tasks.

The detection results of the model on the tomato disease dataset and the paprika disease dataset under different training orders are presented in **Table 7**. Due to different task sequences, we can only compare the model’s performance in detecting known classes after learning 15 tomato diseases. We also compared the model’s memory ability to capture disease patterns and learning ability in cross-species detection tasks such as paprika. The memory ability is reflected in the mAP of previous classes (P), while the learning ability is reflected in the mAP of current classes (C). As expected, learning from more challenging task orders led to a slight performance degradation in the model for all aspects, even though the impact is not significant. This finding serves as a reminder to practitioners that the learning order of models should follow a progression from simpler to more difficult tasks in order to achieve optimal performance.

**Table 7:** Sensitivity analysis on the task training order.

Architecture	PTD	Order	Task 1~3 (Tomato)		Task 4 (cross-species task) (Paprika)					
			K	Δ	P	Δ	C	Δ	K	Δ
R-50-C4	IN1k	Task 1, Task 2, Task 3, Task 4	42.52	–	42.96	–	62.78	–	47.92	–
R-50-C4	IN1k	Task 3, Task 2, Task 1, Task 4	41.37	-1.15	40.8	-2.16	63.03	0.25	46.4	-1.52
R-50-FPN	IN1k	Task 1, Task 2, Task 3, Task 4	44.35	–	44.80	–	65.70	–	50.03	–
R-50-FPN	IN1k	Task 3, Task 2, Task 1, Task 4	42.38	-1.97	42.37	-2.43	58.64	-7.06	46.44	-3.59
R-50-C4	COCO	Task 1, Task 2, Task 3, Task 4	44.24	–	45.90	–	64.24	–	50.48	–
R-50-C4	COCO	Task 3, Task 2, Task 1, Task 4	41.46	-2.78	41.33	-4.57	62.16	-2.08	46.54	-3.94
R-50-FPN	COCO	Task 1, Task 2, Task 3, Task 4	43.58	–	44.92	–	66.31	–	50.27	–
R-50-FPN	COCO	Task 3, Task 2, Task 1, Task 4	42.04	-1.54	41.84	-3.08	65.97	-0.34	47.87	-2.40
R-50-FPN	LVIS	Task 1, Task 2, Task 3, Task 4	47.69	–	47.19	–	69.12	–	52.67	–
R-50-FPN	LVIS	Task 3, Task 2, Task 1, Task 4	43.17	-4.52	43.9	-3.29	68.23	-0.89	49.98	-2.69
R-101-FPN	LVIS	Task 1, Task 2, Task 3, Task 4	46.63	–	47.68	–	72.13	–	53.80	–
R-101-FPN	LVIS	Task 3, Task 2, Task 1, Task 4	44.48	-2.15	44.37	-3.31	68.17	-3.96	50.32	-3.48
X-101-FPN	LVIS	Task 1, Task 2, Task 3, Task 4	48.31	–	48.87	–	71.86	–	54.61	–
X-101-FPN	LVIS	Task 3, Task 2, Task 1, Task 4	47.26	-1.05	46.56	-2.31	71.57	-0.29	52.81	-1.80

We alternately show the performance of different training orders in the same model. Δ represents the differences caused by the order of learning. For the notation of evaluation metrics, please refer to **Table 2**.

'-' indicates that the difference is not applicable to the current situation.

### 4.5 Cross-species detection

**Figure 5:**
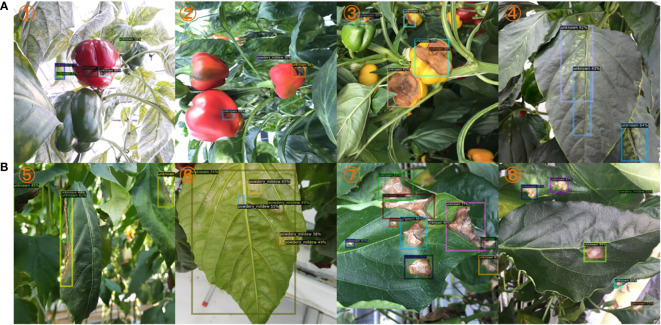
Qualitative results on cross-species detection study. **(A)**. Training on tomato dataset and test on paprika dataset. **(B)**. Training on paprika dataset and test on tomato dataset. The sample number is indicated in the top left corner of each subplot. Best view in color.

Our research has uncovered a fascinating discovery regarding the capabilities of our model, particularly in the context of cross-species disease detection. Despite being trained solely on a dataset of tomato diseases, our model exhibited the remarkable ability to identify and provide an initial assessment of affected regions in paprika fruit diseases. This intriguing finding is illustrated through two specific cases showcased in **Figure 5A**, namely Case 1 and Case 2.

Please note that our model has never been exposed to or trained on any tomato fruit diseases, only leaves, making its performance in detecting paprika fruit diseases all the more intriguing. The fact that the model can generalize its knowledge and effectively apply it to a different species highlights its versatility and potential for cross-species disease detection, which also demonstrates that our method learns the fundamental features of disease.

Furthermore, we conducted a similar experiment in which we trained a separate model using a paprika disease dataset and evaluated its performance on a test dataset consisting of tomato plants. The results were equally compelling. Our paprika-trained model successfully detected pests present on tomato leaves, as demonstrated by Case 8 in **Figure 5B**. This further reinforces the model’s ability to transfer its learned knowledge across species boundaries and adapt it to different contexts.

To provide a comprehensive visualization of the model’s cross-species detection capabilities, **Figure 5** presents qualitative results of these experiments. These visual examples offer a glimpse into the model’s ability to identify diseases and pests in species it has not been explicitly trained on, demonstrating its potential for broader applicability and practical use in real-world scenarios.

## 5 Discussion

The task of object detection is typically divided into two subtasks: classification and localization. In this section, we discuss the limitations of our method in these two subtasks, including open issues. Finally, we present several potential avenues for future research.

### 5.1 Localization

**Figure 6:**
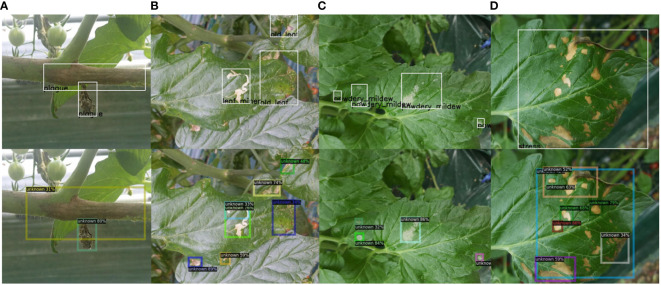
Qualitative results for unknown instances from our dataset. We present four pairs of examples **(A–D)**. The first row displays the image and annotations, while the second row represents our detection results. Best view in color.

Addressing the localization problem of unknown objects is a key challenge in open-world object detection. The main difficulty lies in the lack of prior knowledge about the unknown classes in the model. As a result, it is challenging to directly learn their features and location information from the training data. Our method improved the model’s ability to detect unknown diseases. However, qualitative experimental results showed that the unknown recall is still below 30%. A unified unknown detection evaluation protocol is even more difficult than finding unknown diseases. As shown in **Figure 6**, these unknown detection results are treated as false positive boxes under the current ground truth, even though our model has already localized these suspicious regions.

The controversy surrounding the evaluation criteria for unknown class localization stems from the lack of consistent standards and consensus. This controversy is formed when annotating datasets. Our previous work (Dong et al., 2022) discussed how to efficiently label plant diseases. We believe that different disease symptoms should adopt different labeling strategies, and we verified this scheme’s effectiveness through several experiments. However, these annotation strategies and evaluation schemes were designed for known categories. To the best of our knowledge, no related work discusses the localization of unknown classes in plant disease detection tasks. Additionally, the definition and scope of unknown classes also introduce subjectivity and uncertainty, further contributing to the controversy of evaluation criteria. Therefore, further research and consensus-building are needed to establish consistent and fair evaluation criteria for assessing the localization performance of unknown classes.

### 5.2 Classification

We developed a dynamic open-world detector since plant growth is a dynamic process. However, plant disease dynamics are more complex than we imagined. Some diseases may exhibit different symptoms at different stages of growth, leading to a challenging feature expression. Additionally, different diseases may also exhibit similar symptoms at different stages, which can be due to different pathogens (such as bacteria, fungi, viruses, etc.) or environmental factors. We list some common examples of tomato diseases with similar symptoms:

Yellowing symptoms: Yellowing is a common symptom of many plant diseases, including viral infections, fungal diseases, and nutrient deficiencies. Different pathogens or causes may lead to yellowing of plant leaves or other tissues, but their pathological processes and treatment methods may differ completely.

Leaf spot diseases: Many pathogens can cause similar leaf spot diseases, such as fungal and bacterial leaf spots. They produce similar spots or patches on the leaves, but the pathogens and pathogenic mechanisms behind them are different.

Rotting symptoms: Rotting is a common symptom caused by various diseases or pathogens, including bacterial soft rot, fungal rot, and rotting caused by certain environmental factors. Although they manifest as the decay of plant tissues, the specific causes may be different.

**Figure 7:**
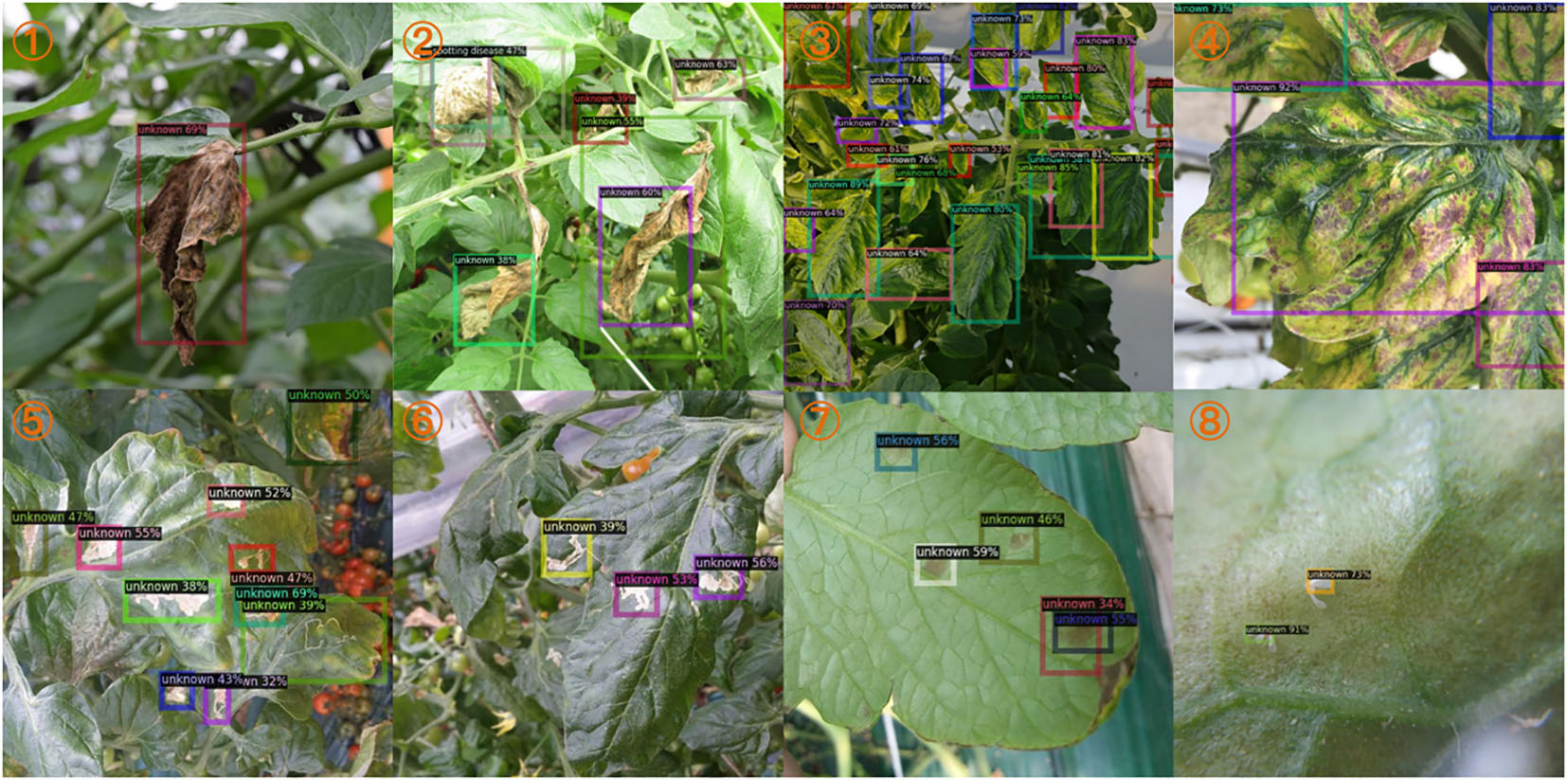
Qualitative results for unknown instances from our dataset. These instances of gray mold should have been detected as unknown. The sample number is indicated in the top left corner of each subplot. Best view in color.

Similar symptoms may also occur in the detection of cross-species diseases. In addition, the leaves of different plants are different in a healthy state. However, diseases may force the leaves of different species to deform to the same symptom at the final state. In this case, even experts also struggle to distinguish them. Therefore, deep learning models may still face the same challenges in accurately differentiating them. **Figure 7** shows some cases with similar symptoms but different species. When testing for diseases on paprika leaves using a model trained on the tomato dataset, all suspicious regions should have been detected as unknown. However, some unknown regions are mistakenly detected as gray mold due to similar symptoms. Although the category is correct, these instances of gray mold are treated as false positives.

These pieces of evidence prove that deep learning models can offer advantages in distinguishing similar disease symptoms but are not infallible. Domain expertise and collaboration with experts remain critical in evaluating and validating the model’s predictions. The model’s success still depends on the availability of quality training data and the complexity of the differentiation task. Another limitation we encountered is the challenge of obtaining additional high-quality datasets for plant disease detection to validate generalizability further. Despite this constraint, we have conducted validation using the COCO dataset to showcase the method’s performance. For more details, please refer to our code repository.

### 5.3 Future works

We present some promising approaches to tackle classification problems. Recently, Du et al. (2022a) introduced spatial-temporal unknown distillation (STUD), a model designed to detect unknown objects in videos by establishing spatial-temporal context. STUD (Du et al., 2022a) utilizes time series features to evaluate the relationship between the current frame and the reference frame, reducing the occurrence of classification errors. In real-world agricultural practices, the same species is commonly planted in one area. Therefore, considering the spatial-temporal context to determine the species category can effectively narrow down the range of disease classifications. Another intriguing direction is utilizing large visual language models (Radford et al., 2021), renowned for their impressive zero-shot detection capabilities, making them highly suitable for identifying unknown categories. A recent study (Wortsman et al., 2022) demonstrated that fine-tuning a large-scale visual language model through weight integration performs well not only on specific downstream tasks but also maintains its ability to recognize unknown targets. Consequently, embedding a large language-vision model into open-world detection tasks has the potential to enhance the model’s robustness. We encourage the community to pay attention to these promising methods and apply them to plant disease detection tasks.

## 6 Conclusions

In this study, we introduced a new paradigm called open world plant disease detector. This novel detection paradigm enables the detection of unknown diseases and allows for the dynamic updating of new knowledge. This paradigm breaks the closed-set, static open-set settings of conventional plant disease detectors. We observed that detectors trained on complex object detection datasets can enhance the detection performance for unknown classes, and the category-agnostic head further improved the recall rate for unknown diseases. Additionally, cross-species disease detection experiments have demonstrated that our model can comprehend the concept of diseases and successfully detect them across different species. Extensive ablation experiments validated the effectiveness of our proposed method. Furthermore, we thoroughly discussed the existing open challenges in plant disease detection and offered insightful perspectives. We strongly encourage researchers and practitioners to address the current challenges that remain.

## Data availability statement

The original contributions presented in the study are included in the article/supplementary files. Further inquiries can be directed to the corresponding authors.

## Author contributions

JD designed the method, performed the experiments, and wrote the manuscript. KH and SY advised in the design of the system and analysed the annotation strategies to find the best method for efficient plant disease detection. AF and DP provided support in the data collection and proofreading article. JY presents some conceptual suggestions and domain knowledge. All authors contributed to the article and approved the submitted version.
